# Dose-painting intensity-modulated proton therapy for intermediate- and high-risk meningioma

**DOI:** 10.1186/s13014-015-0384-x

**Published:** 2015-03-30

**Authors:** Indira Madani, Antony J Lomax, Francesca Albertini, Petra Trnková, Damien C Weber

**Affiliations:** Center for Proton Therapy, Paul Scherrer Institute, Villigen, Switzerland; Ghent University, Ghent, Belgium; Department of Radiation Oncology, University Hospital of Zürich, Zürich, Switzerland

**Keywords:** Intensity-modulated proton beam-therapy, Dose painting, PET, Simultaneous integrated boost, Meningioma

## Abstract

**Background:**

Newly diagnosed WHO grade II-III or any WHO grade recurrent meningioma exhibit an aggressive behavior and thus are considered as high- or intermediate risk tumors. Given the unsatisfactory rates of disease control and survival after primary or adjuvant radiation therapy, optimization of treatment strategies is needed. We investigated the potential of dose-painting intensity-modulated proton beam-therapy (IMPT) for intermediate- and high-risk meningioma.

**Material and methods:**

Imaging data from five patients undergoing proton beam-therapy were used. The dose-painting target was defined using [68]Ga-[1,4,7,10-tetraazacyclododecane tetraacetic acid]– d-Phe^1^,Tyr^3^-octreotate ([68]Ga-DOTATATE)-positron emission tomography (PET) in target delineation. IMPT and photon intensity-modulated radiation therapy (IMRT) treatment plans were generated for each patient using an in-house developed treatment planning system (TPS) supporting spot-scanning technology and a commercial TPS, respectively. Doses of 66 Gy (2.2 Gy/fraction) and 54 Gy (1.8 Gy/fraction) were prescribed to the PET-based planning target volume (PTV_PET_) and the union of PET- and anatomical imaging-based PTV, respectively, in 30 fractions, using simultaneous integrated boost.

**Results:**

Dose coverage of the PTVs_PET_ was equally good or slightly better in IMPT plans: dose inhomogeneity was 10 ± 3% in the IMPT plans vs. 13 ± 1% in the IMRT plans (*p* = 0.33). The brain D_mean_ and brainstem D_50_ were small in the IMPT plans: 26.5 ± 1.5 Gy(RBE) and 0.002 ± 0.0 Gy(RBE), respectively, vs. 29.5 ± 1.5 Gy (*p* = 0.001) and 7.5 ± 11.1 Gy (*p* = 0.02) for the IMRT plans, respectively. The doses delivered to the optic structures were also decreased with IMPT.

**Conclusions:**

Dose-painting IMPT is technically feasible using currently available planning tools and resulted in dose conformity of the dose-painted target comparable to IMRT with a significant reduction of radiation dose delivered to the brain, brainstem and optic apparatus. Dose escalation with IMPT may improve tumor control and decrease radiation-induced toxicity.

## Background

Newly diagnosed WHO grade II and III or any WHO grade recurrent meningioma due to their aggressive behavior [1,2] are considered as high- or intermediate-risk tumors [3]. Surgical excision rarely cures the patient with these challenging tumors and adjuvant treatment is needed. When meningioma is not resectable, primary radiation therapy is an alternative. Meningioma patients can be treated primary or in the adjuvant setting by conventional external beam photon radiotherapy [4], high-precision stereotactic radiosurgery [5], stereotactic fractionated radiotherapy [6], or intensity-modulated radiation therapy (IMRT) [7,8], with the latter treatment modality administrating a substantial dose to non-target tissues. Meningioma can also be treated with protons [9]. Radiation doses ≥60 Gy were identified as a prognostic factor for higher local control, particularly when they were delivered with proton beams [10]. Proton beam-therapy offers superior dose distributional qualities as compared to X- or gamma-rays, as the dose deposition occurs in a modulated narrow zone called the Bragg peak, with no exit dose distal to the target volume. Dose escalation using proton beam-therapy holds the promise of higher local control and survival [11,12]. However, higher radiation doses in large target volumes may increase the risk of radiation-induced toxicity, as reported for stereotactic radiosurgery [13]. One way to overcome this challenge is to escalate the dose in a fraction of the target volume, which is biologically relevant for disease control, applying a dose painting paradigm [14,15]. Dose painting using biological imaging aims at mapping dose distributions to tumor heterogeneity, where radioresistant regions within the tumor would receive higher doses (dose escalation) and radiosensitive regions would be irradiated to conventional or even lower (dose de-escalation) radiation doses.

Meningiomas have increased expression of somatostatin receptors, with the highest expression of subtype 2 (SSTR2A) receptors [16,17]. This has led to suggestions of using positron emission tomography (PET) [68]Ga-labeled somatostatin analogs - [68]Ga-[1,4,7,10-tetraazacyclododecane-*N,N′,N″,N‴*-tetraacetic-acid]-D-Phe^1^-Tyr^3^-octreotide ([68]Ga-DOTATOC) or [68]Ga-[1,4,7,10-tetraazacyclododecane tetraacetic acid]– d-Phe^1^,Tyr^3^-octreotate ([68]Ga-DOTATATE) – to improve tumor detection [18,19] and target volume definition [6,20,21]. [68]Ga-DOTATOC-PET can detect SSTR2A density [22], for which a significant correlation with microvascular density, high histological grade and proliferation-related Ki-67 antigen (Ki67) of the tumor has been reported [23]. Because microvascular density, high histological grade and proliferation are all unfavorable prognostic factors for meningioma [24,25], [^68^Ga]DOTATOC- or DOTATATE-PET-positive regions within the tumor may represent a target for dose escalation, while the rest of the tumor would receive conventional radiation dose. Several target volumes require several dose prescriptions that could be done using either sequential boost or simultaneous integrated boost (SIB), the latter providing the most conformal dose distributions with intensity-modulated techniques [26]. Pencil beam intensity-modulated proton beam-therapy (IMPT) pioneered and currently used in Paul Scherrer Institute (PSI), Villigen, Switzerland allows generation of SIB plans for dose painting aiming at higher dose prescription inside the biological image-based contours. In this study we examined the potential of dose escalation in [68]Ga-DOTATATE-PET-based targets using IMPT as compared to photon-beam IMRT. We evaluated dose coverage of the targets and sparing of organs-at-risk (OARs) in dose-painting plans generated with proton and photon beams.

## Material and methods

### Imaging and target definition

Imaging data of five patients undergoing proton beam-therapy in PSI were used in this study. All patients gave their written informed consent on the use of their data in the institutional board-reviewed study. All patients were immobilized in supine position with a bite block or a mask to minimized head movements. Planning computer tomography (CT) scans (HiSpeed DX/I CT scanner, GE, Chalfont St. Giles, UK) acquired in a treatment planning position with 2-mm slicing were transferred to a RayStation treatment planning system, v. 3.99.0.18 (RaySearch Laboratories AB, Stockholm, Sweden) for co-registration with pre- and post-contrast T1-weighted magnetic resonance (MR) and [68]Ga-DOTATATE-PET/CT scans and delineation of targets and OARs. Dynamic [68]Ga-DOTATATE-PET/CT was performed prior proton beam-therapy at a dedicated GE Discovery VCT PET/CT scanner consisting of a BGO full-ring PET and a 64-slice or spiral CT or GE Discovery DST PET/CT scanner consisting of LYSO full-ring PET and 16-slice spiral CT, both in 3D mode. PET data were reconstructed iteratively with attenuation correction.

Two gross tumor volumes (GTVs) – biological image-based (GTV_PET_) and anatomical image-based (GTV_CT/MR_) – were the results of auto-segmentation of PET-scans using 50% of maximum standardized uptake value (SUV_max_) [27] and manual delineation of the macroscopic tumor visible on CT and/or magnetic resonance (MR) scans, respectively. The union of the 2 GTVs resulted in the GTV_union_. A margin of 5 mm was added to the GTV_union_ to create the union clinical target volume (CTV_union_). The GTV_PET_ and CTV_union_ were isotropically expanded with a 3-mm margin to obtain the PET-based planning target volume (PTV_PET_) and union PTV (PTV_union_), respectively. Delineated OARs included the brainstem itself, the brain, the optic chiasm, ipsilateral optic nerve, retina and lacrimal gland. A 3-mm margin was added to the brainstem, optic chiasm, optic nerve and retina to obtain the respective planning organ-at-risk volumes (PRVs).

### Treatment planning and dose prescription

An in-house developed treatment planning system supporting spot-scanning technology on PSI Gantry 1 [28] was used in IMPT treatment planning. In all but one patient 4 non-coplanar beams were used whilst 1 patient was planned with 3 non-coplanar beams. Selection of gantry and couch angles was based on the PSI Gantry 1 clinical treatment protocol. Initial beam energies were between 138 and 177 MV for each field. Dose computations used a proton ray-casting pencil beam model [29] including heterogeneity corrections [30,31] and allowing 3-dimensional optimization of intensity-modulated proton fields [32]. All IMPT plans were normalized to the mean PTV_PET_ dose.

IMRT treatment planning was performed on the RayStation treatment planning system using a step-and-shoot technique with 6 MV photons of an Elekta Synergy linear accelerator (Elekta, Crowley, UK). All plans were based on 4 individually selected non-coplanar beams. Final dose computations were done with a collapse cone convolution superposition dose engine.

We used SIB in our dose prescription. The prescription doses of 66 Gy (2.2 Gy/fraction) and 54 Gy (1.8 Gy/fraction) were to the PTV_PET_ and PTV_union_, respectively, in 30 fractions. The dose of 66 Gy was equivalent to biologically effective dose (BED2) of 70 Gy in 35 fractions. This dose level matches the radiation dose in the high-risk arm of the phase II European Organization for the Research and Treatment of Cancer (EORTC 22042-26042). We aimed at planning at least 95% of the prescription dose to 95% of the PTV_PET_ and PTV_union_ minus PTV_PET_ and not more than 107% of the prescription dose to 5% in both PTVs. Dose inhomogeneity calculated as (D_2_-D_98_)/D_50_ was set to ≤10% in the PTV_PET_. Less than 5% of the PRVs of the brainstem, optic chiasm and optic nerve were allowed to receive 60 Gy. Less than 5% of the PRV of the retina were allowed to receive 55 Gy. A median dose to the lacrimal gland should not exceed 30 Gy. We used the same treatment planning objectives in IMPT (applying relative biological effectiveness factor of 1.1) and IMRT treatment planning. If a treatment plan did not fulfill dose prescription to the brainstem, optic structures (chiasm, nerve and retina) and lacrimal gland, we would give priority to dose-volume constraints of those OARs aiming at the prescription dose to the CTV. The D_2_ (a surrogate of maximum dose), D_50_, and D_98_ (a surrogate of minimum dose) are dose levels on the dose-volume histograms (DVHs) above which lay 2%, 50%, and 98% of the contoured volume, respectively.

### Statistics

We analyzed individual DVHs and 3-dimensional dose distributions and compared dose-volume metrics of IMPT and IMRT treatment plans using a paired *T*-test considering *p* < 0.05 significant. Statistical analysis was done using a Statistical Package for the Social Sciences (SPSS) software, version 20 (IBM, NY).

## Results

**Figure 1:**
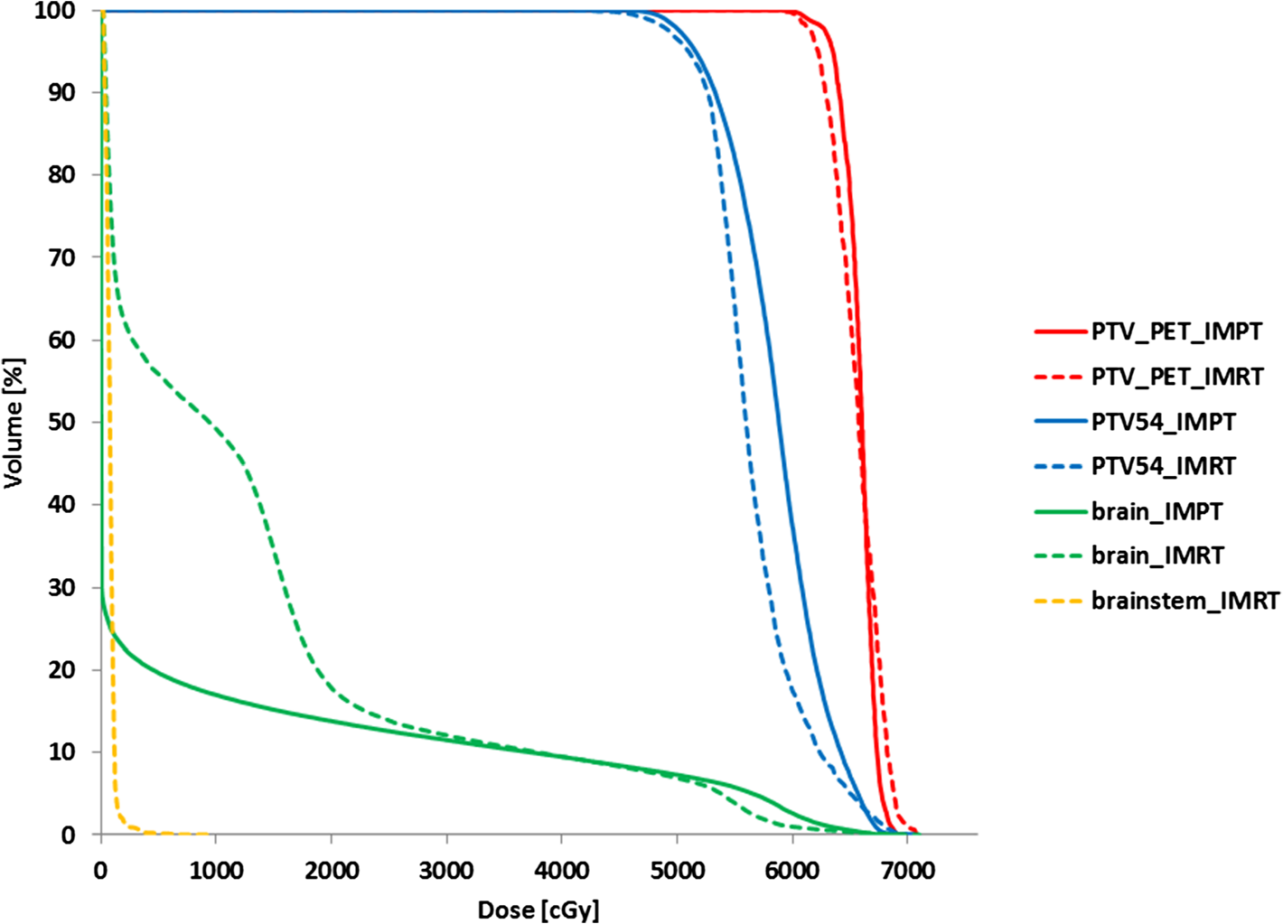
**Dose-volume histograms of intensity-modulated proton-beam therapy (IMPT) in solid and photon intensity-modulated radiation therapy (IMRT) in dash for a patient with a recurrent WHO grade I parasagittal meningioma.** The brainstem in the IMPT plan receives a zero dose.

**Figure 2:**
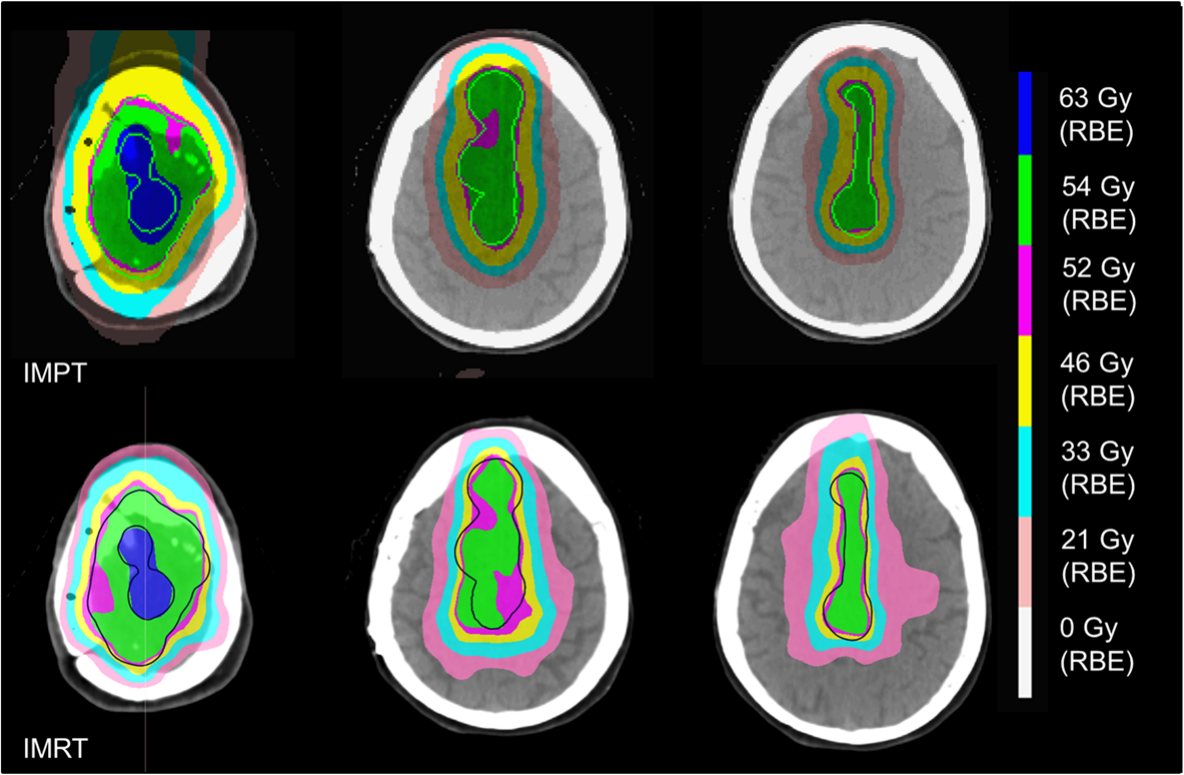
**Dose distributions in the axial plane of the intensity-modulated proton-beam therapy (IMPT) and photon intensity-modulated radiation therapy (IMRT) plans for the same patient.** The union planning target volume (PTV_union_) minus the PET-based PTV is in light green (IMPT) and in blue (IMRT).

Patient characteristics are presented in Table 1. None of the patients were irradiated previously. Four patients had 1 PTV_PET_ and 1 patient had 2 PTVs_PET_. The median volume of the PTV_PET_ and PTV_union_ was 4.3, range 0.31-52.1 cm^3^ and 99.5, range 18.1-199.2 cm^3^, respectively. DVHs and dose distributions of IMPT and IMRT plans for patient 1 are presented in Figure 1 and Figure 2. Compiled dose-volume metrics of all patients are given in Table 2. Treatment-planning objectives were fulfilled in all IMPT and IMRT plans except one case (patient 2), where IMPT failed to spare the ipsilateral lacrimal gland. Dose coverage of the PTV_PET_ did not differ between the two methods with dose inhomogeneity of 10% (IMPT) (range 5.5-15.5%) and 13% (IMRT) (range 7.0-33.2%) on average.

**Table 1:** **Patient characteristics**

**Patient N.**	**Gender**	**Age (year)**	**Tumor location**	**WHO grade**	**Prior surgery**
1	Female	60	Parasaggital	I	2*
2	Female	45	Sphenoid	I	1
3	Female	38	Falx	I	2
4	Male	75	Falx	II	2*
5	Female	64	Falx	II	1

*Combined with embolization.

**Table 2:** **Dose-volume metrics (mean dose ± standard deviation) of intensity-modulated proton beam-therapy (IMPT) and intensity-modulated photon radiotherapy (IMRT) treatment plans**

	**IMPT**	**IMRT**	***p*** **-value**
GTV_PET_			
D98 (Gy_RBE_)	65.3 ± 1.3	63.8 ± 2.8	0.16
D95 (Gy_RBE_)	65.7 ± 1.1	64.4 ± 2.4	0.14
D50 (Gy_RBE_)	67.2 ± 0.9	67.1 ± 1.0	0.67
D2 (Gy_RBE_)	69.0 ± 1.0	69.9 ± 1.9	0.37
PTV_PET_			
D98 (Gy_RBE_)	62.3 ± 1.4	60.1 ± 5.7	0.28
D95 (Gy_RBE_)	63.3 ± 0.7	62.1 ± 2.5	0.19
D50 (Gy_RBE_)	66.2 ± 0.3	65.5 ± 1.2	0.15
D2 (Gy_RBE_)	68.8 ± 0.9	68.6 ± 0.9	0.73
Homogeneity	0.10 ± 0.03	0.13 ± 0.1	0.33
GTV_union_			
D98 (Gy_RBE_)	56.5 ± 2.9	56.0 ± 3.8	0.51
D95 (Gy_RBE_)	57.7 ± 3.0	57.1 ± 3.3	0.44
D50 (Gy_RBE_)	62.3 ± 4.6	61.5 ± 3.6	0.48
D2 (Gy_RBE_)	67.9 ± 1.4	67.9 ± 1.3	0.89
CTV_union_			
D98 (Gy_RBE_)	54.0 ± 2.2	48.7 ± 9.0	0.16
D95 (Gy_RBE_)	55.1 ± 2.1	53.5 ± 3.8	0.14
D50 (Gy_RBE_)	60.1 ± 3.3	58.7 ± 2.5	0.23
D2 (Gy_RBE_)	67.1 ± 1.4	67.1 ± 1.4	0.99
PTV_union_			
D98 (Gy_RBE_)	50.0 ± 13.6	42.0 ± 12.3	0.18
D95 (Gy_RBE_)	51.8 ± 7.4	48.1 ± 6.6	0.22
D50 (Gy_RBE_)	58.3 ± 2.2	57.1 ± 2.0	0.26
D2 (Gy_RBE_)	66.5 ± 1.9	66.6 ± 1.7	0.96
Homogeneity	0.28 ± 0.04	0.43 ± 0.22	0.15
Brain			
D2 (Gy_RBE_)	42.1 ± 24.6	54.3 ± 7.2	0.34
D_mean_ (Gy_RBE_)	26.5 ± 1.5	29.5 ± 1.5	0.001
Brainstem			
D5 (Gy_RBE_)	0.6 ± 1.4	11.0 ± 13.5	0.13
D50 (Gy_RBE_)	0.002 ± 0.0	7.5 ± 11.1	0.02
Optic chiasm			
D5 (Gy_RBE_)	28.1	41.6	-
Ipsilateral optic nerve			
D5 (Gy_RBE_)	59.1	59.4	-

*Abbreviations: GTV* = gross tumor volume; *PTV* = planning target volume; *CTV* = clinical target volume; D_*x*_ = dose level on the dose-volume histograms, above which lay *x*% of the contoured volume.

Doses to the optic chiasm and ipsilateral optic nerve are reported for one patient.

In one patient with the largest PTV_PET_ abutting optic structures dose inhomogeneity was 15% and 33% in the IMPT and IMRT plan, respectively. Dose inhomogeneity achieved with both IMPT and IMRT was still 10% or lower, when there were two dose-painted targets inside the PTV_union_ (patient 3). Dose inhomogeneity was slightly lower in the PTV_union_ in IMPT plans as compared to IMRT – 28% vs. 43%. Nevertheless, D_mean_ of 26.5 ± 1.5 Gy to the brain was significantly less in the IMPT plans than 29.5 ± 1.5 Gy(RBE) in the IMRT plans (*p* = 0.001; Table 2). A significant difference in D_50_ to the brainstem was observed in the IMPT plans - 0.002 ± 0.0 Gy(RBE) vs. 7.5 ± 11.1 Gy in the IMRT plans (p = 0.02; Table 2). There was only one patient in our study (patient 2), who required sparing the optic structures and lacrimal gland. Although dose-volume constraints to the optic nerve and retina were met with IMPT, D_50_ exceeded a 30 Gy limit set for the lacrimal gland - 40.7 Gy(RBE) vs. 14.6 Gy obtained by IMRT. D_5_ to the optic chiasm and brainstem were 1.5 and 10 times less in IMPT plans than in IMRT plans for this patient.

## Discussion

Until the results of the EORTC 22042-26042 and Radiation Therapy Oncology Group 0539 studies [3,33] are available, there is no agreement on what radiation dose level should be prescribed for WHO grade II-III or recurrent meningioma; however, higher doses are believed to be more effective. Several studies examine the benefits of dose escalation – either by increasing the number of fractions with 3-dimensional (3D) conformal photon, photon-proton radiation therapy [3,11,12] or by using a sequential boost with photon 3D-conformal radiation therapy/IMRT [33], IMPT and carbon-ion radiation therapy [34,35]. A few studies demonstrated clinical feasibility of dose escalation combining photon and proton beams [10-12]. Hug et al. showed significant improvement in the local control and survival rates at doses ≥60 cobalt gray equivalent (CGE) in 1.8-2.0 CGE/fraction [10]. Boskos et al. treated 24 patients after surgery for atypical and malignant meningioma to a median total dose of 68 CGE in 1.8-2.0 CGE/fraction [11]. They reported significant association between increase in overall survival and doses >60 CGE. Chan et al. escalated radiation dose to 68.4 and 72 Gy (RBE) in 1.8 Gy (RBE)/fraction in 6 patients with WHO grade II and III meningioma, respectively [12]. Treatment was well tolerated without grade 3 or greater toxicity. In a planning study comparing IMRT, IMPT and carbon-ion radiation therapy using sequential boost, dose escalation in the boosted PTV (median volume 94 ± 46 cm^3^) up to 68 Gy (RBE) in 2 Gy (RBE)/fraction was possible in 2 out of 10 meningioma patients, all planned to 60 Gy (RBE) [34]. Planning to higher doses was limited by tolerance of abutting OARs. We believe that IMPT using SIB can combine many of the benefits of these other treatment approaches, to yield excellent dose coverage of the target, superior sparing of critical OARs, such as the brainstem, brain and optic apparatus (Table 2), shortened treatment time and advantages of hypofractionation.

Into best of our knowledge, this is the first paper evaluating IMPT using SIB for intermediate- and high-risk meningioma [36]. In this study we demonstrated technical feasibility of dose-painting IMPT with the planning tools currently in use at PSI. It is easy to integrate a boost of 2.2 Gy (RBE)/fraction into conventionally - 1.8 Gy (RBE)/fraction - fractionated IMPT. These SIB IMPT plans can be implemented in clinical use immediately after dosimetric verification.

IMPT and IMRT plans have excellent dose coverage of the dose-painted target: dose inhomogeneity is on average 10% and 13%, respectively. However, the high conformity of the IMRT plans has been reached at the cost of larger volumes of the brain and brainstem receiving intermediate- to low doses. This is in contrast to the IMPT plans where there was no or minimal dose to the brain and brainstem (Table 2). There was a significant reduction of D_mean_ to the brain and D_50_ to the brainstem obtained with proton beams for all plans (Table 2). This may be translated into better neurological and cognitive outcomes, particularly if non-involved brain areas responsible for “key” brain functions, e.g., the hippocampus, are included in the IMPT optimization. With the perspective of increased disease control resulting from dose escalation, preservation of patients’ neurocognitive functions and quality-of-life becomes of higher importance.

Nevertheless, the study shows that pencil beam spot size is an important consideration. One of the shortcomings of IMPT planned for PSI Gantry 1 was overdosage of the lacrimal gland, due to large lateral penumbra of the proton beams from that treatment device. Because of the lateral pencil beam width, a small, superficially located gland overlapping the PTV could not be spared as good as with IMRT, though there were higher doses to the brain, brainstem and optic chiasm in IMRT plans. Therefore, using proton beams with smaller lateral width are important to ensure sharper dose gradients and thus better sparing of small OARs in the immediate vicinity to the target [37].

Using [68]Ga-DOTATOC- or DOTATATE-PET in target volume determination leads to target volume modification in approximately 70% of the patients [6,21] with a smaller target volume in 35-50% of the patients [6,20]. One challenge is that the methodology of PET-based target volume segmentation is not standardized and most investigators delineate the target manually by adjusting the window of the PET scans to the GTV visible on CT and/or MR. To avoid drawbacks of manual delineation we have chosen to perform threshold-based auto-segmentation of the GTV_PET_. In absence of consensus guidelines for the cut-off SUV values for PET-based segmentation of meningioma, we have selected 50% SUV_max_ for our PET scans. The resulting PTV_PET_ volumes are quite small, with a median of 4.3 cm^3^. Although no limits have been set on the volume for dose painting, we believe that escalating dose in smaller volumes is a safer strategy. Nevertheless, uncertainties of our delineation strategy warrants further investigation.

## Conclusions

Dose-painting IMPT using SIB is technically feasible with the currently available tools at our institution. IMPT plans resulted in excellent dose coverage of the dose-painted PTV_PET_ and PTV_CT/MR_ at minimal or no dose to the brain, brainstem and optic apparatus that could be translated into improved disease control and radiation-induced toxicity.

## Abbreviations

BED: Bxiologically effective dose
CGE: Cobalt gray equivalent
CT: Computer tomography
DVH: Dose-volume histogram
EORTC: European Organization for the Research and Treatment of Cancer
[68]Ga-DOTATATE: [68] Ga-[1,4,7,10-tetraazacyclododecane tetraacetic acid]– d-Phe^1^,Tyr^3^-octreotate
[68]Ga-DOTATOC: [68] Ga-[1,4,7,10-tetraazacyclododecane-*N,N′,N″,N‴*-tetraacetic-acid]-D-Phe^1^-Tyr^3^-octreotide
GTV: Gross tumor volume
IMPT: Intensity-modulated proton beam-therapy
IMRT: Intensity-modulated radiation therapy
Ki67: proliferation-related Ki-67 antigen
MR: Magnetic resonance imaging
OAR: Organ-at-risk
PSI: Paul Scherrer Institute
PET: Positron emission tomography
PRV: Planning organ-at-risk volume
PTV: Planning target volume
RBE: Relative biological effectiveness
SIB: Simultaneous integrated boost
SSTR2A: Somatostatin receptor subtype 2
SUV: Standardized uptake value
WHO: World Health Organization
